# Evaluation of Tangential Flow Filtration for the Concentration and Separation of Bacteria and Viruses in Contrasting Marine Environments

**DOI:** 10.1371/journal.pone.0136741

**Published:** 2015-08-25

**Authors:** Lanlan Cai, Yunlan Yang, Nianzhi Jiao, Rui Zhang

**Affiliations:** State Key Laboratory of Marine Environmental Science, Institute of Marine Microbes and Ecospheres, Xiamen University (Xiang’an), Xiamen, Fujian, 361102, China; University of Aveiro, PORTUGAL

## Abstract

Tangential flow filtration (TFF), which has been widely adopted to concentrate a diverse array of microbes from water, is a promising method of microbial separation or removal. However, it is essential to select an optimal membrane suitable for the specific filtration application. This study evaluated two different scales of TFF systems for concentrating and separating microbes (including bacteria and viruses) from contrasting marine waters. Among bacteria-size membranes, polyvinylidene difluoride (PVDF) membranes showed higher bacterial recovery, but lower viral permeation efficiencies than polyethersulfone (PES) membranes, regardless of environments and scales of TFF. Estuary samples showed significantly higher percentages of bacterial retention than nearshore and ocean samples. For virus-size membranes, a higher viral recovery and lower sorption was observed for regenerated cellulose membrane than PES membranes in the small-scale TFF. Similar viral recoveries were observed between PES membranes in the large-scale TFF, with higher viral concentrations being observed in estuary samples than in nearshore samples. Deep ocean samples showed the lowest recovery of viruses, which was consistent with observations of bacterial recovery. Synthetically, PVDF may be more suitable for the concentration of bacterial cells, while PES would be a better choice for the collection of viruses. When compared with the PES membrane, regenerated cellulose is better for viral concentration, while PES is recommended to obtain bacteria- and virus-free seawater.

## Introduction

Marine microbes, including bacteria, archaea, viruses and picoeukaryotes, are the most abundant biological group in the ocean [1–3]. These organisms are vital drivers of biogeochemical cycles via their impact on nutrient use and energy flow. Because of their small individual size and biomass, it is necessary to concentrate microbes in environmental samples prior to analysis to obtain sufficient experimental materials. For example, microbes are concentrated to obtain sufficient genomic DNA or RNA for molecular ecological analysis [4,5]. Additionally, the chances of successful isolation and cultivation of novel viruses can be greatly improved through concentration. On the other hand, separation techniques are usually required in biotechnological processes to harvest microbial cells and recover extracellular biometabolites after bacterial fermentation [6]. A variety of methods have been developed for the separation and concentration of microbes. Absorption-elution techniques, which rely on the adsorption of organisms to a solid matrix such as membrane filters, fiberglass, or diatomaceous earth followed by elution into a small volume of buffer, have been used for microbial concentration [7–9]. However, these methods suffer from selective adsorption and inconsistent results that are highly dependent on microbial and water characteristics [10]. Moreover, the buffer used may influence the downstream analysis [11]. Similarly, concentration and separation of the sample with conventional “dead-end” filters and subsequent re-suspension normally result in damage to the microbes [12]. Additionally, large quantities of samples can often not be processed within a reasonable time because of filter clogging. Although ultracentrifugation has been used for the concentration of viral particles, the application of ultracentrifugation is limited by disruption of microorganisms by the high centrifugation speed, small volume capacities, and expensive equipment [13]. Tangential flow filtration (TFF) has emerged as a promising technique for the recovery of diverse microbes in water samples by reducing filter clogging through parallel fluid flows tangent to the filter surface [14,15]. Cross-flow recirculation makes it possible to feed more water (10–1000 L, depending on the system) permeate through the membrane while concentrating particles larger than the pore size in the retentate and decreasing the tendency for microbes to adhere to filter surfaces at the same time. TFF can be equipped with a microporous or ultrafiltration membrane possessing a wide range of pore sizes or molecular cut-offs. This technique has been widely applied for the concentration of cells from cultures in medicinal and biochemical fields [16]. Additionally, TFF has been employed in aquatic microbial ecology studies to concentrate or remove specific populations [17,18]. Although it is a promising technique, TFF has been reported to have variable efficiencies. Previous study of Whitehouse *et al*. showed that organic material and trace metals were recovered by TFF without significant losses [19]. While Winget *et al*. achieved 32–68% bacterial recovery from marine water by TFF in the Chesapeake Bay [20]. Besides that, Colombet *et al*. reported recovery rates of 11–98% for planktonic viruses in < 0.2 μm freshwater samples using tangential flow ultrafiltration (30 kDa cut-off) [21].

In studies of biological oceanography, it is often necessary to quantify samples to enable extrapolation to *in situ* conditions. In such cases, it is important to determine the filtration efficiency because particle recovery will differ with equipment used and sample types. In this study, we evaluated the efficiency of TFF for the concentration and separation of microbes (including bacteria and viruses) from contrasting marine waters. As the performance of a particular TFF system equipped with different membranes in one aquatic environment may not be suitable for another, the efficiencies of small- and large-scale TFF systems were evaluated in estuary, nearshore, and ocean environments with different biological, chemical, and physical characteristics. To the best of our knowledge, this is the first study to comprehensively evaluate the efficiency of TFF for microbes in various types of aquatic environments. The information presented herein can be used as a guide to select the most suitable membrane for a given experiment.

## Materials and Methods

### 2.1. Study sites and sampling

**Fig 1:**
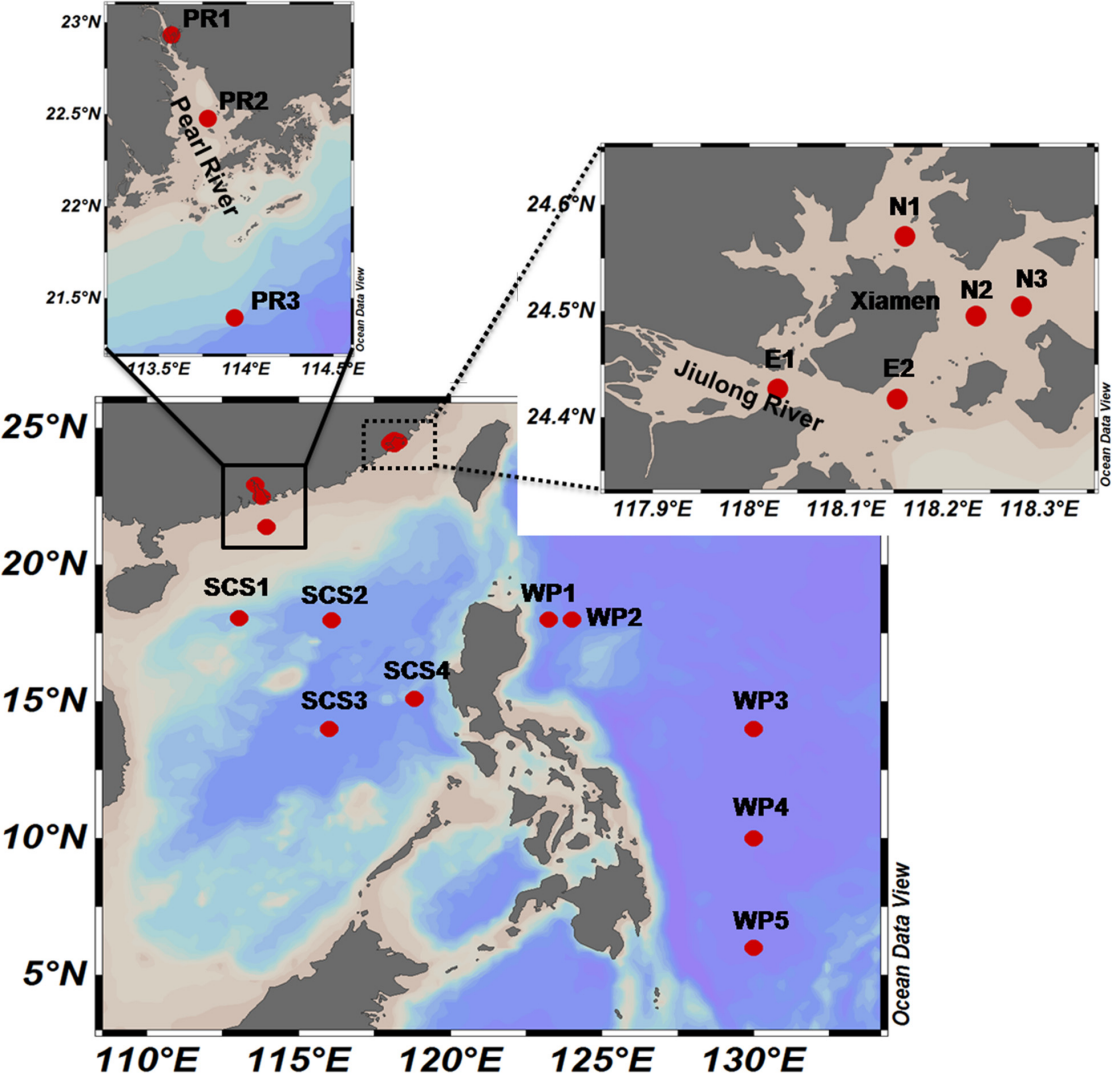
Map showing sampling sites.

Samples were obtained from four sampling areas (Fig 1). (i) Five sites in coastal waters around the Xiamen Island, China. Two of these sites (E1 and E2) were located in the mouth of the Jiulong River, which is considered as a hypereutrophic estuary environment. The remaining sites (N1, N2, and N3) were located in the nearshore environment. (ii) A transect with three sites (PR1–PR3) along the salinity gradient from the Pearl River estuary to the open water of the South China Sea was sampled during a summer cruise. (iii) Four samples were collected from the South China Sea. (iv) Five samples were obtained from the western Pacific Ocean. No specific permissions were required for these locations and sampling activities. The field studies did not involve endangered or protected species. Detailed descriptions of the sampling stations are presented in S1 Table.

### 2.2. Tangential flow filtration

The following terms are used in the text: ‘Feed’ is a sample prefiltered through a 3 μm filter before being used for TFF. ‘Permeate’, also known as filtrate, is the fraction passing through the TFF membrane. ‘Retentate’, also called the concentrate, is the fraction retained by the membrane. The retentate was enriched with microbes that have not passed through the specific membranes.

**Fig 2:**
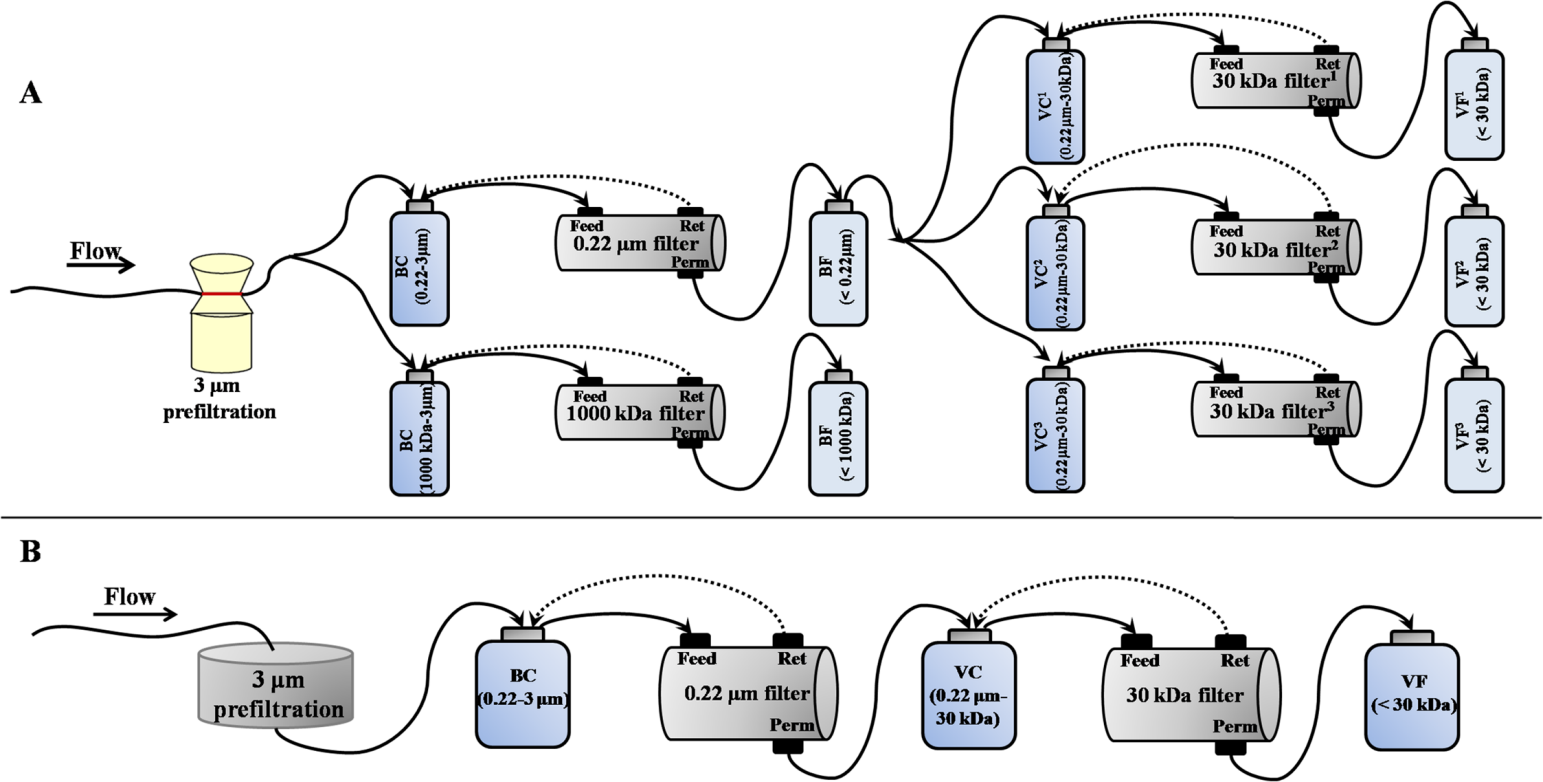
Schematics of small-scale (A) and large-scale (B) TFF systems for the concentration and separation of microbes from water samples. Abbreviations are as follows: Ret, retentate; Perm, permeate; kDa, kiloDalton; BC, bacterial concentration; BF, bacterial filtration; VC, viral concentration; VF, viral filtration; 30 kDa filter^1^, 30 kDa cut-off filter with regenerated cellulose material produced by Millipore; 30 kDa filter^2^, 30 kDa cut-off filter with polyethersulfone material produced by Millipore; 30 kDa filter^3^; 30 kDa cut-off filter with polyethersulfone material produced by Pall.

Two scales of TFF systems were used in the experiment according to the volume of feed water. The large-scale TFF system was equipped with a filtration membrane cassette packed with a total surface area of 0.5 m^2^, which allowed the processing of hundreds of liters of water, while the small-scale system was equipped with a filtration membrane cassette packed with a total surface area of 50 cm^2^, which was used to filter several liters of water. An overview of the experiment is shown in Fig 2.

In the small-scale TFF, two filters with similar pore sizes and different materials (0.22 μm, polyvinylidene difluoride, PVDF, Millipore, Cat. No. PXGVPPC50; 1000 kDa, polyethersulfone, PES, Pall, Cat. No. OA990C12) were initially compared for their efficiencies in three types of environments (estuary, nearshore, and ocean). Next, three 30 kDa cut-off filters with different materials or manufacturers (regenerated cellulose, RCL, Millipore, Cat. No. PXC030C50; PES, Millipore, Cat. No. PXB030A50; PES, Pall, Cat. No. OA030C12) used to concentrate viruses were tested in two contrasting environments (nearshore and ocean).

Two sets of membranes produced by different manufacturers were tested during operation of the large-scale TFF: Millipore (0.22 μm, PVDF, Cat. No. P2GVPPC05; 30 kDa, PES, Cat. No. P2B030A05) and Pall (0.2 μm, PES, Cat. No. PSM20F07; 30 kDa, PES, Cat. No. OS030F07). Tests were simultaneously conducted in the estuarine salinity gradient (Pearl River) in an attempt to encompass the natural range of salinity observed in aquatic environments. Subsequently, in order to evaluate the influence of sample types on the filtration efficiency, replicated large-scale TFF experiments using Millipore membranes (0.22 μm, PVDF, Cat. No. P2GVPPC05; 30 kDa, PES, Cat. No. P2B030A05) were performed for samples from contrasting environments: coastal water of Xiamen, China (nearshore), the Pearl River, China (estuary), and the South China Sea (ocean; including surface and deep-sea).

TFF experiments were carried out as soon as practical after sampling on board. The cartridges were flushed with deionized distilled water, after which the flow through the TFF system was stabilized by recirculating the samples. All samples were passed through a 3 μm filter to remove large particles prior to TFF. For each small-scale TFF membrane test, 2 L initial feed of samples were concentrated to 50 mL with a factor of 40. The initial feed volumes of the large-scale TFF were 100–300 L, which were concentrated to 4 L. During operation, peristaltic pumps were used to ensure constant feed flow rates of 40 ml/min for small-scale TFF system and 1000 ml/min for large-scale TFF system. It took about 1–2 h for each small-scale TFF test and 2–4 h for bacterial filtration and viral recovery of large-scale TFF, respectively. After each experiment, the filtration membrane cassettes were cleaned by flushing with sufficient deionized water, followed by circulation cleaning with 0.1 N NaOH for at least 30 min. The cassettes were then stored in 0.1 N NaOH according to the manufacturer’s recommendations.

### 2.3. Enumeration of microbes

Prior to TFF concentration and after each step, 2 mL aliquots were fixed with a final concentration of 0.5% glutaraldehyde at 4°C for 20 min, then stored at −80°C after snap freezing in liquid nitrogen to determine the microbial abundance. Duplicate samples were collected for microbial enumeration. Viral and bacterial abundances were determined by flow cytometry according to Marie *et al*. [22] and Brussaard [23]. Briefly, the fixed frozen samples were thawed at room temperature, then stained with SYBR Green I (Molecular Probe, Invitrogen). The stained particles were enumerated using a flow cytometer (Epics Altra II, Beckman Coulter) at event rates of 50–200 particles/s (bacteria) or 100–300 particles/s (virus). The analyses of microbial abundance by flow cytometery for each sample were repeated twice. The data acquisition and analysis were performed with the EXPOTM^32^ MultiCOMP software and FCM Express software.

### 2.4. Statistical analysis

Bacterial or viral recovery efficiency was calculated by dividing the number of bacteria or viruses in the retentate by the total number in the prefiltered (feed) water sample, then multiplying the quotient by 100. Bacterial or viral removal efficiency was calculated by one hundred percent minus the percentage of bacteria or viruses in the permeate. The recovery and removal efficiencies for bacteria and viruses were judged to be normally distributed and homoscedastic by SigmaPlot software, version 12.5 (*p* > 0.05, Shapiro-Wilk). A two-tailed *t* test for independent samples was used to determine the differences in recovery efficiency between different TFF systems and marine environments. A *p*-value of < 0.05 was used to indicate statistical significance. The relationships between recovery efficiency, microbial abundance, and environmental factors were investigated using the SPSS software, version 18.

## Results and Discussion

### 3.1. Bacterial filtration efficiency

**Fig 3:**
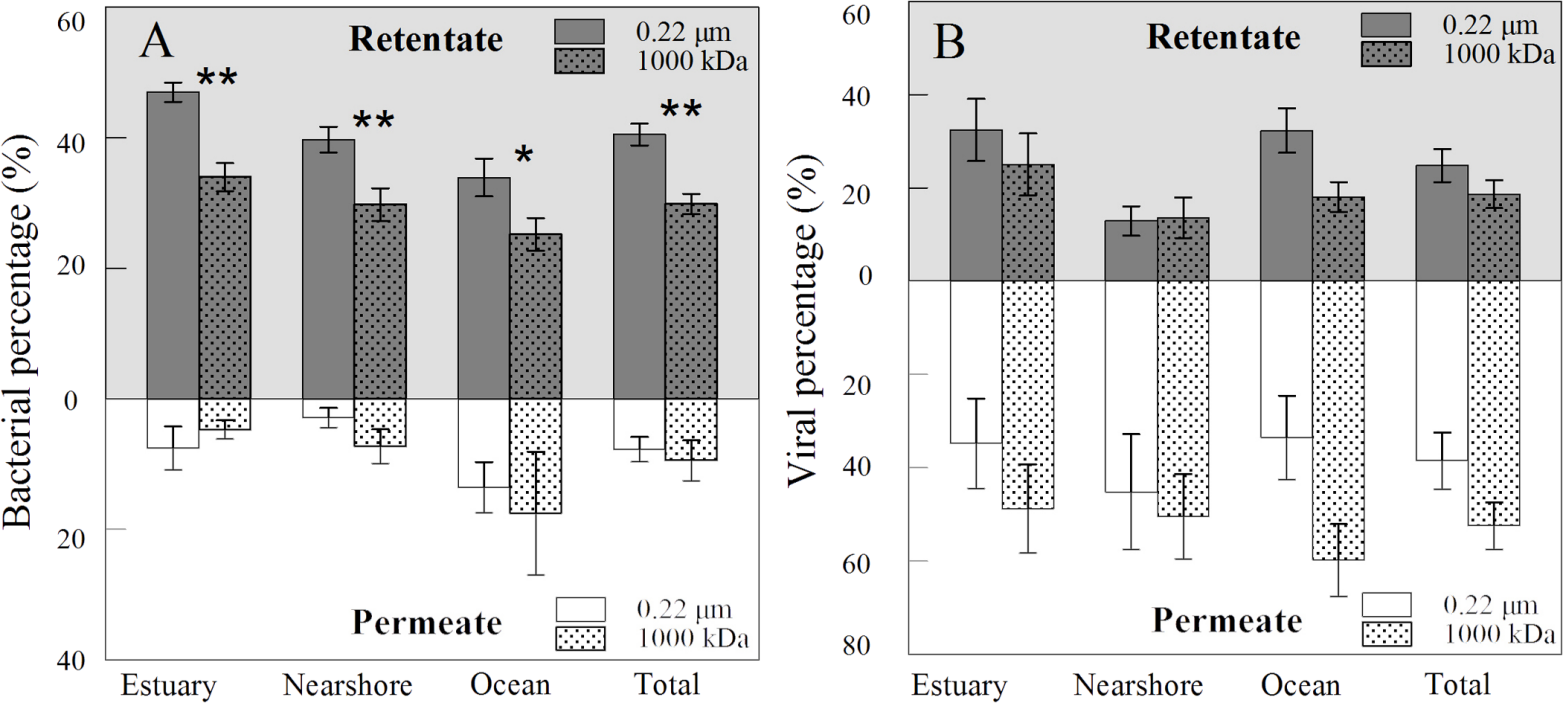
Efficiencies of 0.22 μm and 1000 kDa membranes filtration of bacteria (A) and viruses (B) with small-scale TFF system for contrasting marine environments. Error bars depict the standard error of the mean expressed in percentage. *, *p* < 0.05; **, *p* < 0.01.

Membrane filter with a pore size of 0.22 μm is generally used to remove bacterial cells from samples while enriching cells at the same time. In principle, most bacteria should be retained and concentrated in the retentate, while the permeate contains viruses and some small bacteria that were able to pass through the membranes. In addition, 1000 kDa membrane, which has a pore size approximately equal to 0.1 μm, can be used in similar situations. Although it is often assumed that a smaller membrane pore size results in higher recovery and removal efficiencies, the results of the present study showed that this is not always the case. As shown in Fig 3A, 0.22 μm membrane (PVDF) had a significantly higher (average 40.51%) bacterial recovery in the retentate than 1000 kDa membrane (PES) (average 29.87%) with small-scale TFF for samples from contrasting marine environments (*p* < 0.01, n = 20). In order to evaluate the influence of feed property on the filtration efficiency, small-scale TFF systems equipped with PVDF 0.22 μm and PES 1000 kDa membranes were both tested in three types of environments (estuary, nearshore, and ocean). Generally, the highest bacterial recovery was observed for estuarine environments (average 46.96% and 33.98% for PVDF 0.22 μm and PES 1000 kDa, respectively) and the lowest for oceanic samples (33.92% and 25.18%, respectively) (Fig 3A).

**Fig 4:**
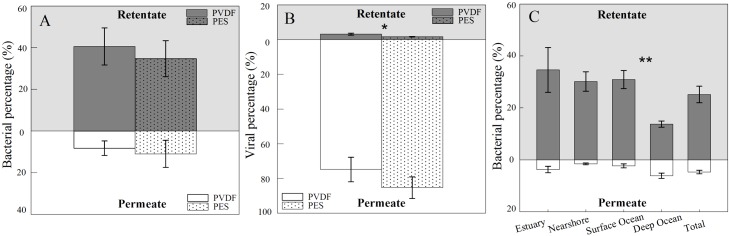
Efficiencies of filtration by bacteria-size membranes for bacteria (A) and viruses (B), and bacterial concentration by Millipore 0.22 μm membrane for samples from contrasting marine environments (C) in large-scale TFF system. Error bars depict the standard error of the mean expressed in percentage. *, *p* < 0.05; **, *p* < 0.01.

Overall, the bacterial recovery efficiency of large-scale TFF was lower than that of small-scale TFF (average 25.16% and 40.51%, respectively). Large-scale TFF with membranes of 0.22 μm and 0.2 μm showed similar patterns at the three stations along the environmental gradient from the Pearl River estuary to the open water of the Northern South China Sea (Fig 4A). On average, 40.55% and 34.64% of bacteria was retained in the retentate for the PVDF and PES membranes, respectively. The 0.22 μm pore-size membrane of large-scale TFF showed 10.33–50.58% bacterial recovery (Fig 4C), with higher bacterial recovery in estuary samples than nearshore samples, which was consistent with observation in the small-scale TFF. While no significant differences were observed between nearshore and surface ocean samples, deep ocean samples showed the lowest recovery of bacterial cells (average 13.74%). Although the investigation for deep-sea bacterial and viral production showed no significant differences between samples measured under decompressed conditions to those measured with *in situ* pressure [24], the environmental changes between *in situ* and experimental conditions for deep-sea microbes, such as temperature and pressure, might alter their physiological status, thus potentially impact recovery efficiency of TFF. In terms of the percentages of bacteria that permeated through the membrane (Figs 3A and 4A), no significant difference was found between the membranes tested for both small- and large-scale TFF systems, which suggests that the removal efficiency was similar for these membranes.

**Fig 5:**
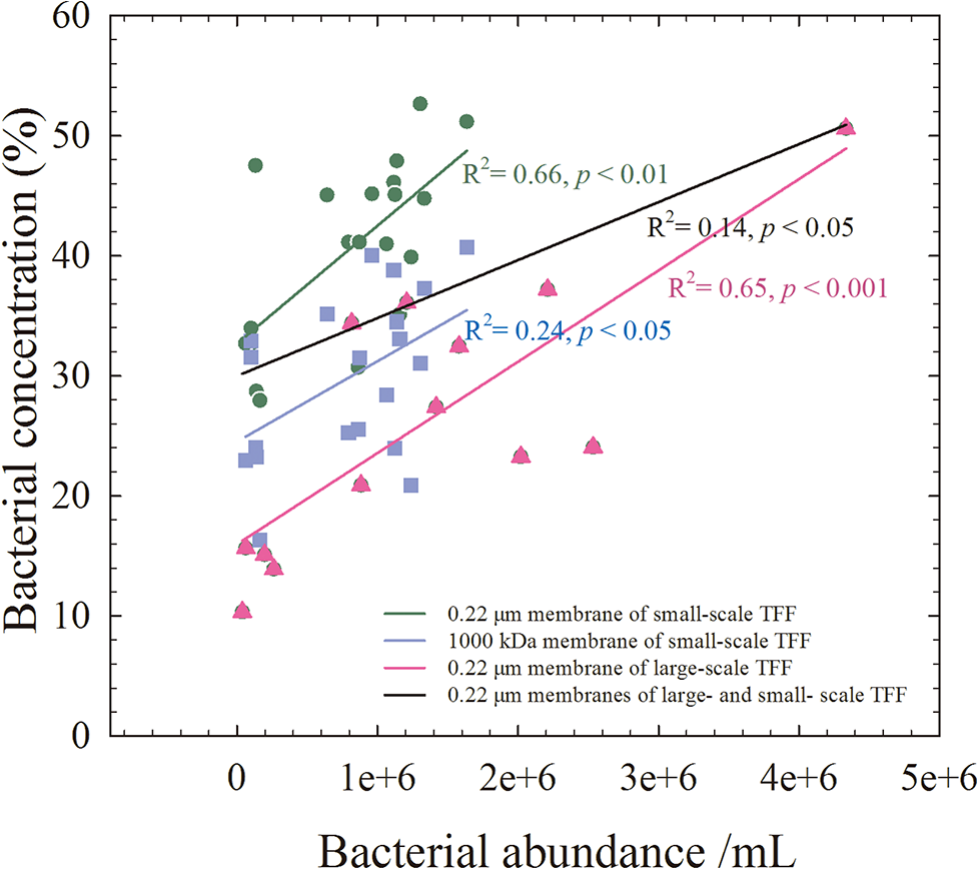
The relationships between bacterial abundance and the efficiency of bacterial recovery

Generally, our data showed that the type of samples significantly impacted the bacterial recovery efficiency for each of the membranes (ANOVA, *p* < 0.05, n = 27), but not the removal efficiency of bacteria (*p* > 0.05). However, bacterial recovery efficiency was not significantly related to sample properties measured in our study, such as salinity, temperature, pH, dissolved oxygen, or nitrate, phosphate, and silicate concentration (ANOVA, *p* > 0.05). A greater bacterial recovery of estuary samples might be the result of a higher level of particles and microbial cells (S1 Table), which may influence the filtration processes. This assumption is supported by a positive correlation between the bacterial recovery and initial bacterial abundance in samples from both small- and large-scale TFF (Fig 5). The presence of abundant bacteria and particles in the feed solution causes steric hindrance to the passage of small bacteria. This may occur because higher abundance of bacteria result in the formation of a dynamic membrane that has a smaller porosity than the original membrane, or because more particles interact with the membrane pore.

### 3.2. Viral filtration efficiency

**Fig 6:**
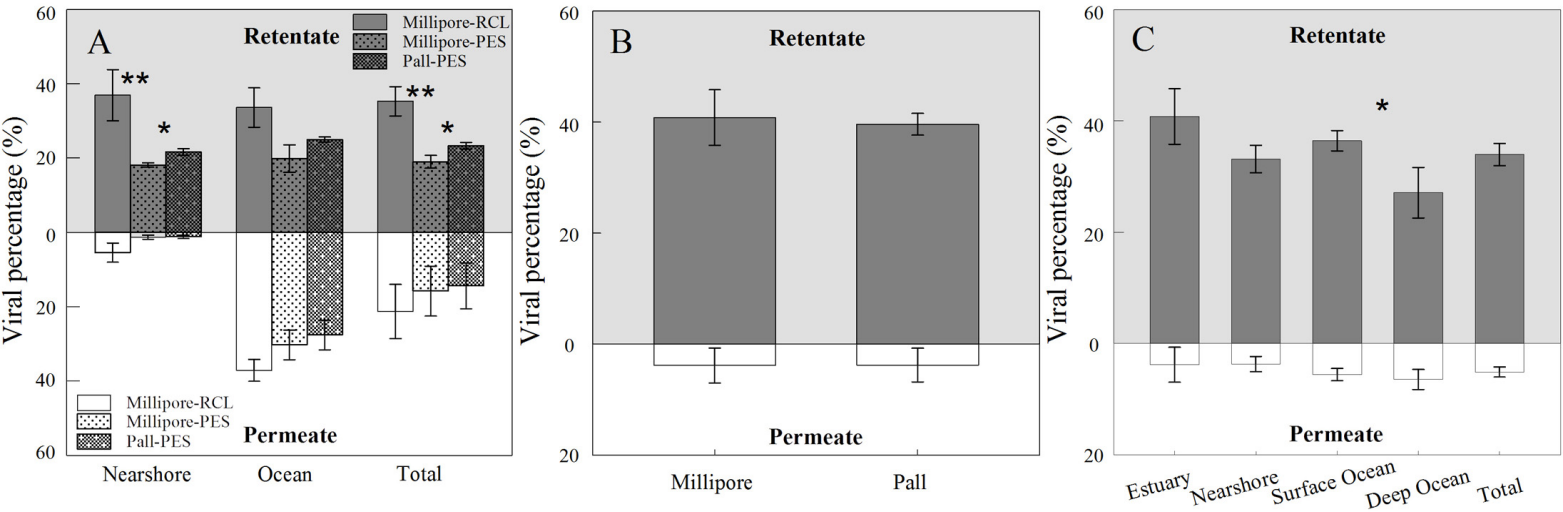
Efficiencies of viral filtration by three 30 kDa membranes for samples from contrasting marine environments in small-scale TFF(A). Comparison of viral filtration by Millipore and Pall 30 kDa cut-off membranes (B) and for samples from contrasting marine environments using Millipore 30 kDa cut-off membrane (C) in large-scale TFF. Error bars depict the standard error of the mean expressed in percentage. *, p <0.05; **, p<0.01.

Removal of bacterial cells using 0.22 μm and 1000 kDa membranes is always the first step to obtain viral concentrations in aquatic viral ecology studies in which low retention, low sorption, and high permeation of viruses are desired. We observed a higher retention and lower permeation of viruses for PVDF membranes than PES membranes in large- and small-scale TFF (Figs 3B and 4B), indicating that PES membranes may be a better choice if more viruses are desired. The filtration capacities of three 30 kDa cut-off membranes with different materials or manufacturers (RCL, Millipore; PES, Millipore; PES, Pall) for viral particles with small-scale TFF were shown in Fig 6A. Generally, 30 kDa cut-off membranes show 12.48–49.57% recovery rates of viruses, which is similar to previous studies [25,26]. The viral recovery efficiency of the RCL membrane (average 35.28%) was higher than that of the two PES membranes (average 19.00% and 23.32%, respectively). In addition, the fraction of viruses that permeate through the membranes was higher for RCL (average 21.26%) than for PES membranes (average 15.76% and 14.36%, respectively), indicating a higher removal rate of viruses with PES membranes. No significant differences were observed in the levels of viruses in the retentate and permeate between the two PES membranes. In large-scale TFF, similar viral recovery efficiencies of 40.82% and 39.60% were observed for the PES membranes manufactured by Millipore and Pall, respectively (Fig 6B). There were also no significant differences in viral filtration (average 3.81% and 3.75%) between these two PES membranes.

**Fig 7:**
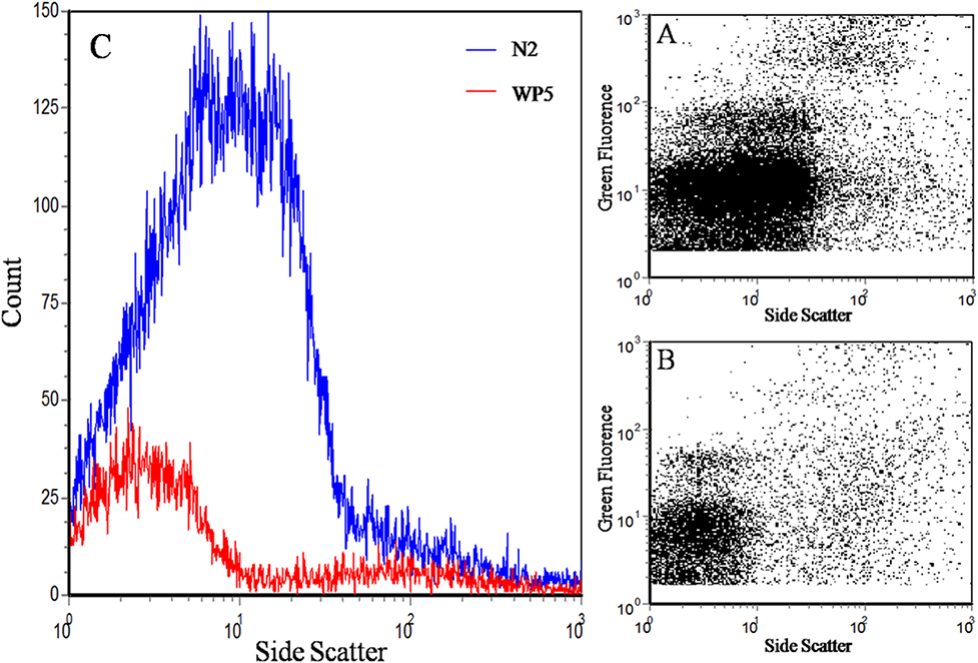
Comparison of samples from Station N2 in Xiamen coastal water (A) and Station WP5 in the western Pacific Ocean (B) in virus-like particle's Side Scatter (C) by flow cytometry.

Spatially, a higher viral recovery was observed in estuary samples (average 40.82%) than nearshore samples (average 33.16%) during large-scale TFF, while no significant differences were observed between nearshore and surface ocean samples. Deep ocean samples showed the lowest of recovery viruses (average 27.12%), which was consistent with the bacterial recovery observed (Fig 6C). For small-scale TFF, the average viral recovery was 25.58% and 26.16% for nearshore and ocean samples, respectively, which was not significantly different. An average of 2.62% of viruses was detected in the viral filtrate of nearshore samples, while 31.64% of oceanic viral particles permeated through the filters (Fig 6A). This might have been because of the smaller size of viruses in the ocean samples, as indicated on the flow cytograms (Fig 7).

### 3.3. Sorption

In the present study, the maximum of 57.67% of recovery rate for microbes is reported, which is similar to the few ones reported [20,21,25,26]. Average 92.57% (n = 56) and 88.50% (n = 35) of removal efficiencies for bacteria and viruses, respectively, are found. In principle, the difference between removal and recovery efficiencies can be attributed to membrane sorption. The high removal efficiencies but relatively low recovery efficiencies for microbes suggests a high sorption of bacteria and viruses on membranes during filtration. As a major problem encountered in TFF and other types of filtration that cannot be completely avoided, sorption was thought to result from the combined effects of different factors; namely, process conditions, sample characteristics, and membrane physico-chemical properties. The physico-chemical properties of membranes that affect adsorption are charge, morphology, and hydrophilicity. Higher recovery and similar removal rates for bacterial cells observed in our study (Figs 3A and 4A) suggested that the PVDF membrane had a lower sorption (52.69%) for bacterial cells than the PES membrane (60.03%) (*p* < 0.05, n = 23), which was likely due to the hydrophilic properties of PVDF membrane. These findings are consistent with the previous results that PES membranes were much more severely fouled than PVDF membranes owing to their relatively high roughness and hydrophobicity [27]. For viral filtration, sorption accounted for 43.46% and 63.77% for RCL membrane and PES membranes, respectively. Similar results were obtained in a previous study of freshwater systems, showing greater carbon sorption on PES than RCL [28]. This can be explained by the greater hydrophobic interactions of samples with PES and charge interactions with RCL [29]. The significant difference in sorption of viruses seen for the nearshore and ocean samples in our study (Fig 6C) was most likely the result of the different amounts of substances present in these samples. Hoffmann *et al*. reported that sorption of carbon appears to be better at lower conductivity, which can be explained by the much lower sorption of membranes in the ocean samples than the nearshore samples that support high levels of substances [28].

## Conclusion

In aquatic microbial ecology, TFF has been widely applied for size fractionation of specific populations depending on the scientific needs. It should be noted, however, that commercially available TFF membranes are designed primarily for water purification, biomedical and food processing, and not for the fine characterization of natural microbial assemblages, which are highly diluted and of widely varying size in sea waters with complex chemical composition. Accurate comparison remains difficult because very few studies where aquatic microbes are concentrated by TFF have provided filtration efficiencies. The present study simultaneously evaluated the concentration and removal efficiencies of various TFF membranes in contrasting marine environments. The results of our evaluation of TFF membranes for marine samples can be summarized as follows:

1. Membrane materials and sample characteristics impact the efficiencies of tangential flow filtration for microbes.
2. PVDF membranes show higher recovery and lower sorption of bacterial cells than PES membranes, while their removal efficiencies for bacterial cells are similar.
3. PES membranes allow more viral particles permeate than PVDF membranes in the process to remove bacterial cells, and RCL membranes concentrate more viral particles than PES membranes.

## Supporting Information

S1 Table The information of sampling stations. (DOCX)
