# Supplementary material for: Evaluation of Tangential Flow Filtration for the Concentration and Separation of Bacteria and Viruses in Contrasting Marine Environments
Source: PLoS One. 2015 Aug 25;10(8):e0136741. doi: 10.1371/journal.pone.0136741 (PMC4549332; doi:10.1371/journal.pone.0136741)
Supplement: S1 Table — (DOCX) [file pone.0136741.s001.docx]

**Supporting information**

**S1 Table. The information of sampling stations**

| Environment | Station | Longitude (E) | Latitude (N) | Sampling time | Depth (m) | Sample replication number | | | |
| --- | --- | --- | --- | --- | --- | --- | --- | --- | --- |
|  |  |  |  |  |  | Small-scale TFF | | Large-scale TFF | |
|  |  |  |  |  |  | 0.22 μm-1000 kDa membrane tests | 30 kDa membrane tests | Millipore-Pall membrane tests | Millipore membrane tests |
| Nearshore | N1 | 118°14'05.43" | 24°29'44.11" | 29-May-2013 | 1 | 2 | 3 |  |  |
|  | N2 | 118°16'54.56" | 24°30'17.00" | 29-May-2013 | 1 | 2 |  |  |  |
|  | N3 | 118°09'40.33" | 24°34'13.99" | 29-May-2013 | 1 | 2 |  |  |  |
| Estuary | E1 | 118°01'47.06" | 24°25'36.71" | 29-May-2013 | 1 | 2 |  |  |  |
|  | E2 | 118°09'12.28" | 24°25'02.40" | 29-May-2013 | 1 | 2 |  |  |  |
|  | PR1 | 113°34'21.60" | 22°54'06.67" | 29-Jul-2013 | 1 |  |  | 1 |  |
|  | PR2 | 113°46'48.12" | 22°21'27.48" | 27-Jul-2013 | 1 |  |  | 1 |  |
| Ocean (surface) | PR3 | 113°56'08.90" | 21°12'42.00" | 4-Aug-2013 | 1 |  |  | 1 |  |
|  | WP1 | 123°14'51.11" | 17°59'48.05" | 8-Dec-2012 | 1 | 2 |  |  |  |
|  | WP2 | 123°59'57.25" | 18°00'03.28" | 9-Dec-2012 | 1 | 2 | 2 |  |  |
|  | WP3 | 129°59'44.92" | 14°01'12.74" | 12-Dec-2012 | 1 | 2 | 1 |  |  |
|  | WP4 | 130°00'05.79" | 9°59'59.46" | 11-Dec-2012 | 1 | 2 |  |  |  |
|  | WP5 | 130°00'57.13" | 5°59'47.53" | 6-Dec-12 | 1 | 2 |  |  |  |
|  | SCS1 | 113°03'07.92" | 18°03'19.00" | 16-Apr-2012 | 1 |  |  |  | 1 |
| Ocean (Deep) | SCS2 | 116°05'12.00" | 14°15'17.22" | 13-Apr-2012 | 3000 |  |  |  | 1 |
|  | SCS3 | 116°09'21.54" | 17°58'58.01" | 21-Apr-2012 | 4000 |  |  |  | 1 |
|  | SCS4 | 118°49'45.00" | 15°06'03.00'' | 18-Apr-2012 | 2000 |  |  |  | 1 |

| Temperature (℃) | Salinity | Conductivity (S/m) | pH | Dissolved oxygen (mg/L) | Turbidity (FTU) | Nitrate concentration (μmol/L) | Phosphate concentration (μmol/L) | Silicate concentration (μmol/L) |
| --- | --- | --- | --- | --- | --- | --- | --- | --- |
|  |  |  |  |  |  |  |  |  |
|  |  |  |  |  |  |  |  |  |
| 29.20 | 29.27 | 4.89 | 7.75 | 6.31 | Na | 64.44 | 4.44 | 51.38 |
| 28.70 | 29.82 | 4.94 | 7.72 | 6.77 | Na | 66.82 | 3.18 | 51.61 |
| 28.40 | 30.50 | 4.71 | 7.77 | 6.89 | Na | 28.30 | 1.07 | 27.45 |
| 28.00 | 16.49 | 2.08 | 7.51 | 6.15 | Na | 92.94 | 2.62 | 85.82 |
| 28.20 | 23.74 | 4.57 | 7.54 | 6.58 | Na | 56.84 | 1.66 | 45.75 |
| 29.01 | 0.14 | 2.50 | 6.17 | 1.20 | Na | Na | Na | Na |
| 28.00 | 20.87 | 2.90 | 8.02 | 6.70 | Na | Na | Na | Na |
| 29.09 | 34.06 | 5.10 | 8.25 | 6.60 | Na | Na | Na | Na |
| 28.50 | 30.91 | 5.08 | Na | Na | 0.19 | Na | Na | Na |
| 28.53 | 27.75 | 4.64 | Na | Na | 0.11 | Na | Na | Na |
| 31.85 | 27.79 | 5.13 | Na | Na | 0.06 | Na | Na | Na |
| 29.85 | 30.54 | 5.15 | Na | Na | 0.13 | Na | Na | Na |
| 29.17 | 26.94 | 4.54 | Na | Na | 0.06 | Na | Na | Na |
| 25.38 | 34.19 | 5.24 | Na | Na | Na | Na | Na | Na |
| 2.36 | 34.62 | 3.20 | Na | Na | Na | Na | Na | Na |
| 2.44 | 34.62 | 3.24 | Na | Na | Na | Na | Na | Na |
| 2.48 | 34.61 | 3.18 | Na | Na | Na | Na | Na | Na |

Na=Not available
